# Liver Assessment in Patients with Ataxia-Telangiectasia: Transient Elastography Detects Early Stages of Steatosis and Fibrosis

**DOI:** 10.1155/2023/2877350

**Published:** 2023-03-11

**Authors:** H. Donath, S. Wölke, V. Knop, U. Heß, R. P. Duecker, J. Trischler, T. Poynard, R. Schubert, S. Zielen

**Affiliations:** ^1^Department for Children and Adolescents, Division of Allergology, Pulmonology and Cystic Fibrosis, University Hospital Frankfurt, Goethe University, Frankfurt, Germany; ^2^Department of Internal Medicine 1, University Hospital Frankfurt, Goethe University, Frankfurt, Germany; ^3^Assistance Publique-Hôpitaux de Paris, Pitié-Salpêtrière Hospital, Hepatology Department, Frankfurt, Germany

## Abstract

**Background:**

Ataxia-telangiectasia (A-T) is a rare autosomal-recessive multisystem disorder characterized by pronounced cerebellar ataxia, telangiectasia, cancer predisposition, and altered body composition. Liver diseases with steatosis, fibrosis, and hepatocellular carcinoma are frequent findings in older patients but sensitive noninvasive diagnostic tools are lacking.

**Objectives:**

To determine the sensitivity of transient elastography (TE) as a screening tool for early hepatic tissue changes and serum biomarkers for liver disease.

**Methods:**

Thirty-one A-T patients aged 2 to 25 years were examined prospectively from 2016–2018 by TE. In addition, we evaluated the diagnostic performance of liver biomarkers for steatosis and necroinflammatory activity (SteatoTest and ActiTest, Biopredictive, Paris) compared to TE. For calculation and comparison, patients were divided into two groups (<12, >12 years of age).

**Results:**

TE revealed steatosis in 2/21 (10%) younger patients compared to 9/10 (90%) older patients. Fibrosis was present in 3/10 (30%) older patients as assessed by TE. We found a significant correlation of steatosis with SteatoTest, alpha-fetoprotein (AFP), HbA1c, and triglycerides. Liver stiffness correlated significantly with SteatoTest, ActiTest, HbA1c, and triglycerides.

**Conclusion:**

Liver disease is a common finding in older A-T patients. TE is an objective measure to detect early stages of steatosis and fibrosis. SteatoTest and ActiTest are a good diagnostic assessment for steatosis and necroinflammatory activity in patients with A-T and confirmed the TE results.

## 1. Introduction

Ataxia-telangiectasia (A-T) is a rare, autosomal-recessive multisystem disorder characterized by progressive cerebellar ataxia, telangiectasia, immunodeficiency, and cancer predisposition [1–5]. Apart from the name-giving brain involvement, the disease also affects the lungs, endocrine system, and liver [6–13]. A-T-associated liver disease is an upcoming health issue that emerges in the second decade of life [7, 14]. Autopsy reports in A-T patients showed liver-specific pathological findings like nonalcoholic steatohepatitis (NASH), liver cirrhosis, and hepatocellular carcinoma (HCC) [7, 15–18].

Hepatopathy in A-T is usually mild and does not lead to a limitation of the synthesis or detoxification function of the liver [7]; however, up to 92.9% of older A-T patients are affected [14]. Liver disease certainly belongs to the complex phenotype of premature aging [19], which also includes insulin resistance (IR), diabetes mellitus type 2 [6, 20], and dyslipidemia and thus leads to an incomplete metabolic syndrome. All these factors naturally favor fatty remodeling of the liver with a consecutive increase in liver enzymes. In addition, a few severe cases of liver failure and HCC have been published as case reports on A-T patients [15, 17].

One of the central aims of modern hepatology is the search for noninvasive diagnostic procedures for presymptomatic liver diseases in at-risk patient groups. In order to prevent severe courses and identify critical patients at an early stage, sensitive, fast, and minimally invasive screening tools are needed to monitor the course of the disease [21, 22]. Currently, a liver biopsy is the gold standard for assessing the severity of nonalcoholic fatty liver disease (NAFLD), NASH, and the stage of liver fibrosis. Nevertheless, limitations and complications include invasiveness, severe bleeding, sampling error, and pneumothorax [23].

Transient elastography (TE) is a precise, noninvasive method to determine the extent of fibrosis and the degree of fatty degeneration of the liver [24]. In contrast to biopsy, it is painless and noninvasive. In addition, it records a multiple of the parenchyma. The classification into fibrosis and steatosis stages is objective in comparison to normal sonography, as it is carried out using fixed cutoff values [22, 24, 25]. Measurements are reproducible and independent of the user [25, 26].

In this cross-sectional study, we prospectively evaluated liver assessment by TE and liver scores (FibroMax) for A-T-associated liver disease to predict the extent of liver disease and identify at-risk patients for severe disease courses.

## 2. Patients and Methods

The current study is a prospective, cross-sectional, clinical, single-center trial.

### 2.1. Patients

From November 2016 to May 2018, 31 patients with a clinically and/or genetically confirmed diagnosis of A-T aged between two and 25 years were included in the study (Table 1). Malignancy and clinical and laboratory-associated infections were defined as the exclusion criteria.

The Ethics Committee of University Hospital Frankfurt approved the trial (Reference No. 504/15). The study was registered at clinicaltrials.gov (NCT03357978). One study visit was conducted. Written consent was obtained from all patients and/or caregivers. The study was conducted according to the ethical principles of the Declaration of Helsinki and regulatory requirements and the code of Good Clinical Practice.

We compared patients <12 years of age (group 1) with those ≥12 years (group 2).

### 2.2. Transient Elastography

The examination was performed with FibroScan® (Echosens, Paris, France). The examination probe is formed by a vibration generator and an ultrasonic probe (3.5 MHz) aligned on the same axis. The vibration generator oscillates at a frequency of 50 Hz, which leads to shear waves in the liver tissue. The speed of propagation of this shear wave correlates directly with liver stiffness and therefore with the extent of fibrosis. The result of this liver stiffness measurement (LSM) is given in kilopascals (kPa) [25].

The interpretation of the measurement results was based on the limit values of a study on mixed hepatopathy by Fraquelli et al. [26]. A distinction was made between three fibrosis stages: *F* ≥ 2 = pronounced fibrosis, *F* ≥ 3 = severe fibrosis, and *F*4 = cirrhosis.

At the same time, the controlled attenuation parameter (CAP) was measured using the same signals. The attenuation of the ultrasound signal (3.5 MHz) in the liver is measured in dB/m. The attenuation correlates with the degree of liver steatosis [27]. The different stages of steatosis were defined as follows: *S* ≥ 1 = steatosis in 11–33% of hepatocytes, *S* ≥ 2 = steatosis in 34–66% of hepatocytes, and *S* = 3 steatosis in 67–100% of hepatocytes. The cutoff values proposed by Karlas et al. in 2017 were used [28].

The examination was performed by an experienced physician in the supine position and maximum abduction of the right arm through a right intercostal space. In order to perform a standardized measurement, patients were asked to fast for at least 4 hours before the examination. A success rate of at least 60% or the interquartile range below 30% of the median measurement result was considered necessary.

The accuracy of the measurement may be reduced in obesity or ascites.

### 2.3. Liver Biomarkers and Liver Scores

FibroMax® (BioPredictive, Paris, France) is a noninvasive blood test for NAFLD screening that has been validated against liver biopsies [29] and is recommended by European guidelines [30]. FibroMax is composed of three different tests (SteatoTest, ActiTest, and FibroTest) for the assessment of steatosis, necroinflammatory activity, and fibrosis, respectively. The serum parameters such as *α*2-macroglobulin, haptoglobin, apolipoprotein A1, bilirubin, gamma-glutamyl transferase (GGT), alanine aminotransferase (ALT), aspartate aminotransferase (AST), fasting glucose, cholesterol, and triglycerides, as well as gender, age, weight, and height, were recorded for the calculation of the liver score FibroMax.

The FibroMax data were calculated by BioPredictive (Paris, France) using a patented algorithm.

The FibroTest results were interpreted using the METAVIR score from *F*0 to *F*4: *F*0 = no fibrosis, *F*1 = portal fibrosis without septa, *F*2 = few septa, *F*3 = many septa without cirrhosis, and *F*4 = cirrhosis.

The steatosis test results were interpreted using the steatosis score from *S*0 to *S*4: *S*0 = no steatosis, *S*1 = mild steatosis, *S*2 = moderate steatosis, *S*3 = pronounced steatosis, and *S*4 = severe steatosis.

The ActiTest results were interpreted using the METAVIR score from *A*0 to *A*3: *A*0 = no necroinflammatory activity, *A*1 = mild activity, *A*2 = moderate activity, and *A*3 = severe activity. Data were interpreted according to Poynard et al. [31, 32].

In addition to liver biomarkers, alpha-fetoprotein (AFP), hemoglobin A1c (HbA1c), complete lipid profile (including cholesterol, high-density lipoprotein (HDL) and low-density lipoprotein (LDL) cholesterol, and triglycerides) and C-reactive protein (CRP) as inflammatory markers were determined.

### 2.4. Statistical Analysis

For statistical analysis, GraphPad Prism 5.01 (GraphPad Software, Inc.) was used. Values are presented as arithmetic means with standard deviation (SD). For comparisons between the two study groups, the two-tailed Mann–Whitney *U* test was applied. Correlations were analyzed by Spearman's correlation coefficient. *p* values  ≤ 0.05 were considered significant.

## 3. Results

In a study period of 19 months (November 2016 to May 2018), 31 patients with A-T were examined. Patients' characteristics are shown in Table 1. The age distribution ranged from 2 to 25 years (mean age: 10.7 years). The patients were divided into the two groups for evaluation. Twenty-one patients were <12 years (group 1), and ten patients were ≥12 years (group 2). No patient had a history of infectious hepatitis or was taking hepatotoxic drugs regularly.

### 3.1. Transient Elastography

**Figure 1:**
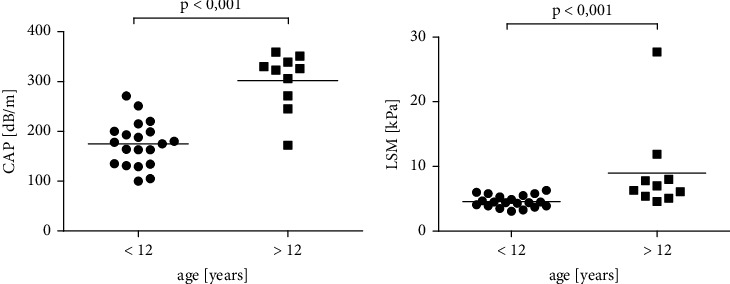
(a) Steatosis assessed by TE. (b) Liver stiffness assessed by TE. Steatosis and liver stiffness are more pronounced in older patients. *p* < 0.001.

**Figure 2:**
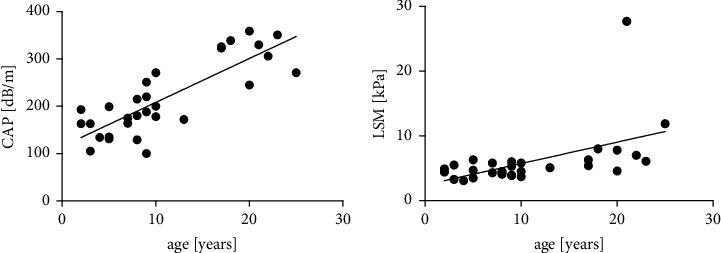
(a) Correlation of steatosis with age. (b) Correlation of liver stiffness with age. Steatosis and liver stiffness progress with age. *p* < 0.001.

On average, 16.5 measurements were performed with a mean success rate of 74%. TE revealed steatosis in 2/21 (10%) of younger patients. In both cases, grade 2 steatosis was present. Fibrosis was not evident in any of younger patients. In comparison, steatosis was detectable in 9/10 (90%) (2 (20%) patients with grade 2 steatosis and 7 (70%) patients with grade 3 steatosis), and fibrosis was observed in 3/10 (30%, median age: 21 years) of older patients. Of these, one patient (10%, aged 20 years) had pronounced fibrosis and the other two patients (20%, aged between 21 and 25 years) had liver cirrhosis (fibrosis stage 4). These results are summarized in Supplementary Table 1. The corresponding LSM (group 1: 4.5 ± 0.93 kPa vs. group 2: 8.9 ± 6.9 kPa; *p* < 0.001) and CAP (group 1: 174.7 ± 45.08 bD/m vs. group 2: 302.2 dB/m ± 57.68 dB/m; *p* < 0.001) of the older group were significantly increased compared to those of the younger group (Figures 1(a) and 1(b)). Furthermore, a positive correlation of the values with age was shown (LSM: *r* = 0.59, *p* < 0.001; CAP: *r* = 0.82, *p* < 0.0001) (Figures 2(a) and 2(b)). The average age for steatosis in group 2 was 20.3 ± 2.7 years.

In addition, CAP and LSM correlated with ALT, AST, GGT, AFP, HbA1c, and triglycerides. There was a significant correlation of steatosis (CAP values) with ALT (*r* = 0.77, *p* < 0.0001), AST (*r* = 0.39, *p* < 0.05), GGT (*r* = 0.83, *p* < 0.0001), AFP (*r* = 0.42, *p* < 0.05), HbA1c (*r* = 0.59, *p* < 0.01), and triglycerides (*r* = 0.74, *p* < 0.00001). LSM correlated significantly with ALT (*r* = 0.53, *p* < 0.01), AST (*r* = 0.42, *p* < 0.05), GGT (*r* = 0.67, *p* < 0.0001), HbA1c (*r* = 0.63, *p* < 0.001), and triglycerides (*r* = 0.62, *p* < 0.001).

### 3.2. Serum Biomarkers and Liver Scores

A complete data set for biomarkers was available for 30 patients. The results of FibroMax are shown in Table 2. Significantly lower values for group 1 concerning SteatoTest and ActiTest (SteatoTest: *p* < 0.0001; ActiTest: *p* < 0.001) were calculated, showing a normal function in group 1 and mild to moderate dysfunction in group 2. FibroTest did not show significant differences between the two groups, with normal to slightly elevated levels in all patients.

The results of SteatoTest indicated steatosis in 8/10 (80%) patients of group 2, whereas no patient in group 1 was affected. Three of ten (30%) older patients had mild steatosis, 2/10 (20%) had mild to moderate steatosis, 2/10 (20%) had moderate steatosis, and one patient (10%) had pronounced steatosis. SteatoTest had a significant correlation with age, LDL-HDL ratios, CRP, and necroinflammatory activity (ActiTest) (age: *r* = 0.74, *p* < 0.0001; LDL-HDL ratio: *r* = 0.79, *p* < 0.0001; CRP: *r* = 0.51, *p* < 0.01; and ActiTest: *r* = 0.89, *p* < 0.0001), as shown in Table 3.

**Figure 3:**
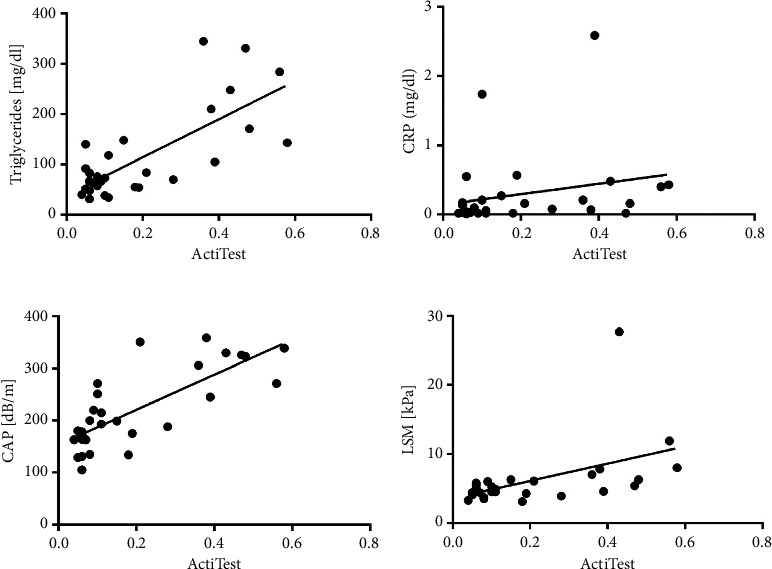
(a) Correlation of ActiTest with triglycerides, (b) correlation of ActiTest with CRP, (c) correlation of ActiTest with CAP, and (d) correlation of ActiTest with LSM.

The ActiTest for the assessment of necroinflammatory activity showed a minimal activity level of stage A0-1 in 3/20 (15%) patients of group 1 and a pathological result in 9/10 (90%) of older patients. One older patient had stage A0-1, 6/10 (60%) patients had stage A1-2, and 2/10 (20%) patients had stage A2, i.e., moderate necroinflammatory activity. ActiTest correlated significantly with triglycerides, CRP, CAP, and LSM, as shown in Figures 3(a)–3(d) and Table 3 (triglycerides: *r* = 0.61, *p* < 0.001; CRP: *r* = 0.41, *p* < 0.05; CAP: *r* = 0.77, *p* < 0.0001; and LSM: *r* = 0.53, *p* < 0.01). In addition, there was a significant correlation with age and the LDL-HDL ratio (age: *r* = 0.8, *p* < 0.0001; LDL-HDL ratio: *r* = 0.8, *p* < 0.0001).

FibroTest did not show a significant difference between the two patient groups. Six of twenty (30%) younger patients had fibrosis according to FibroTest. 4/20 (20%) patients had stage 0-1, one patient had stage 1-2, and one patient had stage 2. In the older group, 3/10 (30%) patients had stage 0-1 and 2/10 (20%) had stage 1-2 fibrosis. This means that a total of 5/10 (50%) patients were affected. The correlations of SteatoTest and ActiTest are shown in Table 3.

The number of patients in each category of SteatoTest, ActiTest, and FibroTest is shown in Supplementary Table 2.

### 3.3. Examination of Metabolic Biomarkers, Inflammation, and AFP

Table 4 shows lipid parameters and HbA1c. Four of ten older patients had type 2 diabetes. No difference was found between the two groups in total cholesterol. However, when broken down into HDL and LDL cholesterol, the older group showed significantly lower HDL cholesterol and significantly higher LDL cholesterol values (HDL cholesterol: *p* ≤ 0.001; LDL cholesterol: *p* ≤ 0.05).

The LDL cholesterol values of the two groups were within the normal range. HDL cholesterol was lower in 8/20 (40%) younger patients and in all older patients (100%). There was a significant difference in the LDL-HDL ratio between the two patient groups (*p* ≤ 0.0001). Triglycerides were significantly increased in group 2 (*p* < 0.0001). Five of ten (50%) older patients had values above the normal range. In group 1, the triglyceride values were all within the normal range. In addition, there was a significant correlation of triglycerides with age (*r* = 0.66, *p* ≤ 0.0001).

As expected, AFP was elevated in all patients. However, there was a significant correlation of AFP values with age (*r* = 0.54, *p* < 0.01).

For CRP, no significant difference between the two groups was found.

## 4. Discussion

A-T is a life-limiting systemic disease clinically characterized by neurodegeneration, radiosensitivity, increased risk of malignancy, immunodeficiency, failure to thrive, and hepatopathy [1, 3, 9–11, 14, 33]. To date, the clinical significance of liver disease is unclear, but up to over 90% of patients develop an elevation of liver enzymes with advancing age, which is associated with a high degree of fatty degeneration and sometimes fibrosis of the liver tissue [14].

To the best of our knowledge, the present study is the first prospective trial addressing noninvasive procedures to characterize liver disease in A-T and relate outcomes of TE to liver biomarkers and liver scores. Our results demonstrate for the first time that 10% of younger patients as opposed to 90% of older patients have liver steatosis. In line with this finding, higher degrees of steatosis were present in the older group. Pronounced fibrosis was found in 3/10 (30%) older patients. In two patients, cirrhosis was already present according to TE. We found a significantly higher necroinflammatory activity (ActiTest) in the older patient group. Necroinflammation can be defined as the immune response to necrosis [34]. Therefore, ActiTest is a good marker for the progression of liver disease to steatosis and apoptosis with increased necroinflammatory activity. The presence of necroinflammatory activity, which also correlates with the CRP value, indicates NASH, which according to our data mainly affects older patients. In summary, SteatoTest and ActiTest are a suitable diagnostic assessment for steatosis and necroinflammatory activity in patients with A-T and have confirmed the TE results.

FibroTest did not show a significant difference between the two patient groups or a significant correlation with age or the results of TE, most likely since FibroMax is not licensed below the age of 14 years due to the physiological increase of some of the serum markers used for calculation.

We were also able to show a significant correlation of AFP with steatosis (CAP). However, the mechanism has not been elucidated so far. AFP is mainly known as a tumor marker for HCC. Serum AFP may also be elevated by germ cell tumors, viral hepatitis, liver fibrosis, and neurodegenerative diseases such as A-T [35–37]. Among other mechanisms, tumor suppressor p53 acts as a repressor on the AFP gene during development and regeneration of the liver [38–40]. Via the reduced activation of p53 due to the absence of the ataxia-telangiectasia-mutated (ATM) kinase, an increased expression of AFP could thus occur in A-T [41]. A mutation of p53 is also frequently found in HCC [42], which could be a possible explanation for an increase in AFP. In contrast to CAP, however, no significant correlation of LSM with AFP was found. The missing correlation of AFP with the LSM could thus be explained by a loss of functional liver tissue in cirrhosis.

Dyslipidemia is common in older A-T patients [6, 8]. While triglycerides and LDL cholesterol were significantly higher, HDL cholesterol was significantly lower in the older patient group. In addition, there was a significant correlation of triglycerides with age. The association between elevated liver enzymes and dyslipidemia has been described before [6]. We could also show a significant correlation of HbA1c and triglycerides with CAP and LSM, emphasizing the effects of metabolic risk factors for liver tissue remodeling. Diabetic metabolism leads to fatty liver remodeling due to hyperglycemia and hypertriglyceridemia and thus increases the risk of NASH. In addition, the described alterations in cholesterol and triglyceride levels in patients indicate an increased atherosclerotic risk profile [43]. They could also play an important role in the development of steatosis, through the accumulation of fat in the liver [44]. The increased influx of free fatty acids to the liver leads to an increased synthesis of triglycerides and very low-density lipoprotein (VLDL), as well as a reduced synthesis of HDL cholesterol [45].

ATM is induced by the accumulation of fat and thus elevates oxidative stress in the liver [46] and acts as an activator of p53, which in turn activates the p53 upregulated modulator of apoptosis (PUMA) [47]. PUMA is a crucial player in steatosis and apoptosis in hepatocytes [48]. Since hepatocyte apoptosis correlates with the severity of NASH and the stage of fibrosis [49], it can be assumed that steatosis related apoptosis is partly responsible for the progression of liver disease [46]. In the absence of ATM, alternative signaling pathways must be activated, which ultimately cause fibrotic remodeling and death of functional liver tissue.

Fibrosis results from connective tissue remodeling due to chronic inflammation of the liver tissue [50, 51], e.g., in response to steatosis and apoptosis [46]. The progression of fibrosis to cirrhosis of the liver then appears to develop through repetitive phases of inflammation and the subsequent reparative immune response [52]. This leads to a loss of functional liver tissue, which is replaced by scar tissue [50, 53].

ATM can be activated directly by oxidative stress, even independently by double-strand breaks in DNA [54]. The absence of ATM leads to low antioxidant capacity [55]. As a result, macromolecules, lipids, and DNA are exposed to permanent oxidative stress and damage it causes. Similar results have been shown for NAFLD and in particular NASH, where oxidative stress and lipid peroxidation are also elevated [56]. For example, hepatocytes with excess fat are particularly susceptible to oxidative stress and DNA damage [57]. This may be related to the fact that saturated free fatty acids promote the formation of ROS, thereby inducing apoptosis and inflammation [58]. It is also known that oxidative stress increases with the severity of liver disease [57]. Oxidative stress and resulting inflammation appear to play a major role in the development and progression of liver disease. A link between hepatopathy in A-T and the already known increased oxidative stress levels in patients therefore seems very likely.

The type of liver involvement in A-T is not yet defined, as the published cases with severe portal hypertension causing ascites and varices had no evidence of cirrhosis at a liver biopsy [15, 16, 18]. It seems that whether these complications are to be attributed to steatosis/NASH leading to portal hypertension in the absence of cirrhosis [59] or to vascular liver disease/porto-sinusoidal vascular disorder (PSVD) is still open for discussion [60]. In this view, clinicians have to consider the value of a liver biopsy, especially in a research context where little is known about liver disease in A-T (i.e., the diagnosis of PSVD can be histological). The rate of adverse events in the modern era is negligible, and the information one can obtain is precious.

The study has some limitations. Due to the size of the study population and the monocentric study design, no general statements can be made. There are no corresponding ultrasound reports that can be compared with the TE results. This is a major limit, not only because it could have shown how liver disease (i.e., fibrosis staging) would have been underestimated by ultrasound only, but also because signs of portal hypertension (i.e., splenomegaly, dilated portal vein, and collaterals) could have been detected and correlated with the absence/presence of liver cirrhosis at TE. In addition, the application of spleen stiffness would have been useful to address more specifically the severity of portal hypertension, as is complementary to LSM [61], and the spleen stiffness measurement (SSM)/LSM ratio could be informative in these cases [62].

Additionally, the examination methods used in this study are not yet established practice in pediatrics, so there are no universally accepted cutoff values for children, but several studies have highlighted the value of TE with CAP [63–66]. The proposed cutoff values are largely consistent with those for adults, which were used in this study. Also FibroTest could show its diagnostic value in some pediatric studies and could distinguish between severe fibrosis and absence of fibrosis [67]. To the best of our knowledge, there are no pediatric studies on FibroMax to date.

Due to the mildness of liver disease, validation of TE by biopsy (gold standard) was not performed. Although CAP is not recommended as a standard tool for stratification of hepatic steatosis [68], there are data showing a good correlation with other steatosis markers, and our data also support this. Another limitation is the lack of a healthy control group to compare the results. As only one TE measurement was performed in each patient, no interobserver comparison is possible. For detection of steatosis by TE, larger cohorts and multicenter studies are needed in order to validate the clinical application.

## 5. Conclusion

Premature aging, low-grade inflammation, oxidative stress, dyslipidemia, and IR as common features of A-T contribute to the development of NAFLD. Liver disease in the context of A-T should be monitored regularly in order to prevent long-term consequences, such as NASH, cirrhosis, and HCC. TE and liver scores are a well reproducible, noninvasive method detecting early stages of liver disease. SteatoTest and ActiTest are useful to assess steatosis and necroinflammatory activity in patients with A-T and have confirmed the TE results.

## Figures and Tables

**Table 1:** Patients' characteristics.

Parameter	Group 1 (*n* = 21)	Group 2 (*n* = 10)	*p* value
Sex	9♀/12♂	5♀/5♂	
Age (years)	6.5 ± 2.8	19.6 ± 3.5	<0.0001
CAP (dB/m)	174.7 ± 45.1	302.2 ± 57.7	<0.001
LSM (kPa)	4.6 ± 0.9	9 ± 6.9	<0.001
AFP (ng/mL)	313.4 ± 267.2	540.8 ± 275.8	<0.05
CRP (mg/dL)	0.2 ± 0.4	0.5 ± 0.8	n.s.
AST (U/L)	37.8 ± 7.9	49.8 ± 15.2	<0.05
ALT (U/L)	25.1 ± 9.6	71.6 ± 25.8	<0.001
GGT (U/L)	13.2 ± 4.5	123.7 ± 99.6	<0.0001

Mean values ± SD are shown; n.s. = not significant.

**Table 2:** Results of FibroMax.

Parameter	Group 1 (*n* = 20)	Group 2 (*n* = 10)	*p* value
SteatoTest	0.02 ± 0.02	0.47 ± 0.24	<0.0001
ActiTest	0.1 ± 0.06	0.39 ± 0.16	<0.001

Mean values ± SD are shown.

**Table 3:** Correlation of SteatoTest and ActiTest.

Parameter	Correlated parameter	*R*	*p* value
SteatoTest	Age	0.74	<0.0001
	CAP	0.69	<0.0001
	LSM	0.51	<0.01
	Triglycerides	0.71	<0.0001
	LDL/HDL	0.79	<0.0001
	HbA1c	0.67	<0.001
	ActiTest	0.89	<0.0001
	CRP	0.51	<0.01

ActiTest	Age	0.80	<0.0001
	CAP	0.77	<0.0001
	LSM	0.53	<0.01
	Triglycerides	0.61	<0.001
	LDL/HDL	0.80	<0.0001
	CRP	0.41	<0.05

**Table 4:** Metabolic biomarkers.

Parameter	Group 1 (*n* = 21)	Group 2 (*n* = 10)	*p* value
Cholesterol (mg/dL)	178.1 ± 36.3	190.9 ± 32.4	n.s.
HDL (mg/dL)	62.3 ± 20.4	35.1 ± 6.4	<0.001
LDL (mg/dL)	106.5 ± 23.5	123.8 ± 25.1	<0.05
LDL-HDL ratio	1.9 ± 0.6	3.7 ± 1	<0.0001
Triglycerides (mg/dL)	66.5 ± 34.3	200.4 ± 98.8	<0.0001
Diabetes type 2	0	4 (40%)	
HbA1C (%Hb)	4.8 ± 0.4	5.7 ± 0.6	<0.0001

Mean values ± SD are shown.

## Data Availability

The datasets used and/or analyzed during the current study are available from the corresponding author on reasonable request.

## Abbreviations:

A-T: Ataxia-telangiectasia
NASH: Nonalcoholic steatohepatitis
BMI: Body mass index
HCC: Hepatocellular carcinoma
IR: Insulin resistance
NAFLD: Nonalcoholic fatty liver disease
HbA1c: Hemoglobin A1c
TE: Transient elastography
CF: Cystic fibrosis
CFLD: Cystic fibrosis liver disease
CRP: C-reactive protein
LSM: Liver stiffness measurement
CAP: Controlled attenuation parameter
GGT: Gamma-glutamyl transferase
ALT: Alanine aminotransferase
AST: Aspartate aminotransferase
AFP: Alpha-fetoprotein
HDL: High-density lipoprotein
LDL: Low-density lipoprotein
SD: Standard deviation
ATM: Ataxia-telangiectasia-mutated
VLDL: Very low-density lipoprotein
PUMA: P53 upregulated modulator of apoptosis
PSVD: Porto-sinusoidal vascular disorder.

## Additional Points

*At a Glance Commentary*. Scientific knowledge on the subject: The majority of older A-T patients suffer from liver disease. This is the first prospective measurement of transient elastography in a larger A-T cohort. *What This Study Adds to the Field*. Transient elastography is a new, noninvasive, and reproducible technique that measures liver stiffness and detects early stages of NASH and cirrhosis in A-T patients.

## Supplementary Materials

Supplementary Materials Supplementary Table 1: an overview of the distribution of fibrosis and steatosis stages detected by TE. Supplementary Table 2: the distribution of the stage of liver involvement measured by ActiTest, SteatoTest, and FibroTest.
