# Supplementary material for: Liver Assessment in Patients with Ataxia-Telangiectasia: Transient Elastography Detects Early Stages of Steatosis and Fibrosis
Source: Can J Gastroenterol Hepatol. 2023 Mar 11;2023:2877350. doi: 10.1155/2023/2877350 (PMC10024628; doi:10.1155/2023/2877350)
Supplement: Supplementary Materials — Supplementary Table 1: an overview of the distribution of fibrosis and steatosis stages detected by TE. Supplementary Table 2: the distribution of the stage of liver involvement measured by ActiTest, SteatoTest, and FibroTest. [file 2877350.f1.docx]

**Suppl. Table 1: Distribution of fibrosis and steatosis stage detected by TE**

| Fibrosis | n= | Steatosis | n= |
| --- | --- | --- | --- |
| F0-1 | 25 | S0 | 19 |
| F2 | 3 | S1 | 0 |
| F3 | 0 | S2 | 4 |
| F4 | 2 | S3 | 7 |

TE was available for 30 patients.

**Suppl. Table 2: Distribution of FibroMax**

| \| SteatoTest \| n= \| FibroTest \| n= \| ActiTest \| n= \| \| --- \| --- \| --- \| --- \| --- \| --- \| \| S0 \| 22 \| F0 \| 26# \| A0 \| 22° \| \| S1 \| 5* \| F1 \| 3^ \| A1 \| 6+ \| \| S2 \| 2 \| F2 \| 1 \| A2 \| 2 \| \| S3 \| 1 \| F3 \| 0 \| A3 \| 0 \| |
| --- | --- | --- | --- | --- | --- | --- | --- | --- | --- | --- | --- | --- | --- | --- | --- | --- | --- | --- | --- | --- | --- | --- | --- | --- | --- | --- | --- | --- | --- | --- |

Complete data set for the FibroMax was available for 30 patients.

*two patients S1-2; #seven patients F0-1; ^three patients F1-2; °four patients A0-1; +six patients A1-2
